# Content analysis of Advance Directives completed by patients with advanced cancer as part of an Advance Care Planning intervention: insights gained from the ACTION trial

**DOI:** 10.1007/s00520-019-04956-1

**Published:** 2019-07-05

**Authors:** Marieke Zwakman, J. J. M. van Delden, G. Caswell, L. Deliens, F. Ingravallo, L. J. Jabbarian, A. T. Johnsen, I. J. Korfage, A. Mimić, C. Møller Arnfeldt, N. J. Preston, M. C. Kars, On behalf of the ACTION consortium, A. van der Heide, I. J. Korfage, J. A. C. Rietjens, L. J. Jabbarian, S. Polinder, P. F. A. Billekens, J. J. M. van Delden, M. C. Kars, M. Zwakman, L. Deliens, M. N. Verkissen, K. Eecloo, K. Faes, K. Pollock, J. Seymour, G. Caswell, A. Wilcock, L. Bramley, S. Payne, N. Preston, L. Dunleavy, E. Sowerby, G. Miccinesi, F. Bulli, F. Ingravallo, G. Carreras, A. Toccafondi, G. Gorini, U. Lunder, B. Červ, A. Simonič, A. Mimić, H. Kodba Čeh, P. Ozbič, M. Groenvold, C. Møller Arnfeldt, A. Thit Johnsen

**Affiliations:** 1 Julius Center for Health Sciences and Primary Care, University Medical Center Utrecht, Stratenum 6.131, PO Box 85500, 3508 GA Utrecht, The Netherlands; 2 Faculty of Medicine and Health Sciences, School of Health Sciences, University of Nottingham, Nottingham, UK; 3 End-of-life Care Research Group, Vrije Universiteit Brussel, Brussels, Belgium; 4 Department of Public Health and Primary Care, Ghent University, Ghent, Belgium; 5 Department of Medical and Surgical Sciences, University of Bologna, Bologna, Italy; 6 Department of Public Health, Erasmus MC, University Medical Center, Rotterdam, The Netherlands; 7 Department of Palliative Medicine, Bispebjerg and Frederiksberg Hospital, Research Unit, Copenhagen, Denmark; 8 Department of Psychology, University of Southern Denmark, Copenhagen, Denmark; 9 University Clinic for Respiratory and Allergic Diseases Golnik, Golnik, Slovenia; 10 Department of Public Health, University of Copenhagen, Copenhagen, Denmark; 11 International Observatory on the End-of-Life Care, Lancaster University, Lancaster, LA1 4YG UK

**Keywords:** Advance Directive, Advance Care Planning, Cancer, Content analysis, End of life

## Abstract

**Purpose:**

Writing an Advance Directive (AD) is often seen as a part of Advance Care Planning (ACP). ADs may include specific preferences regarding future care and treatment and information that provides a context for healthcare professionals and relatives in case they have to make decisions for the patient. The aim of this study was to get insight into the content of ADs as completed by patients with advanced cancer who participated in ACP conversations.

**Methods:**

A mixed methods study involving content analysis and descriptive statistics was used to describe the content of completed My Preferences forms, an AD used in the intervention arm of the ACTION trial, testing the effectiveness of the ACTION Respecting Choices ACP intervention.

**Results:**

In total, 33% of 442 patients who received the ACTION RC ACP intervention completed a My Preferences form. Document completion varied per country: 10.4% (United Kingdom), 20.6% (Denmark), 29.2% (Belgium), 41.7% (the Netherlands), 61.3% (Italy) and 63.9% (Slovenia). Content analysis showed that ‘maintaining normal life’ and ‘experiencing meaningful relationships’ were important for patients to live well. Fears and worries mainly concerned disease progression, pain or becoming dependent. Patients hoped for prolongation of life and to be looked after by healthcare professionals. Most patients preferred to be resuscitated and 44% of the patients expressed maximizing comfort as their goal of future care. Most patients preferred ‘home’ as final place of care.

**Conclusions:**

My Preferences forms provide some insights into patients’ perspectives and preferences. However, understanding the reasoning behind preferences requires conversations with patients.

**Electronic supplementary material:**

The online version of this article (10.1007/s00520-019-04956-1) contains supplementary material, which is available to authorized users.

## Introduction

An Advance Directive (AD) provides a framework for patients to document thoughts regarding future medical care and treatment, to ensure that their wishes and preferences can be followed if they become unable to make their own decisions [1]. Although ADs can be helpful in maintaining the quality of a patient’s end of life [2, 3], the majority of people do not have an AD, mainly due to a lack of knowledge of ADs or because an AD is considered unnecessary now [4, 5]. Consequently, the use of ADs in clinical practice remains low [4–10]. Advance Care Planning (ACP) conversations can be effective to increase the rate of completed ADs [11–13]. Therefore, the completion of ADs is no longer seen as self-contained, but rather as a component of ACP. This perspective is reflected in recently developed definitions of ACP that include the opportunity to document wishes for future care and treatment as part of the ACP process [14, 15].

Currently, most ADs concern do-not-resuscitate orders, or a durable power of attorney for healthcare, and they often involve expressions of concrete treatment preferences [16–18]. However, if ADs are part of the ACP process, it may be helpful if they also include information on patients’ values, beliefs and more general wishes. This provides a context for understanding the patient whenever healthcare professionals and relatives are to make decisions on behalf of patients who are not able to speak for themselves. To our knowledge, there is only one study that has investigated the content of ADs covering a broader range of topics [19]. This study showed that patients with haematological malignancies described aspects related to medical treatments or actions, effective pain treatment and personal messages for their family in their ADs. What patients describe in a more comprehensive AD in the context of a guided ACP conversation has not yet been investigated. Consequently, we do not know whether patients provide in-depth information on their preferences in their ADs after having participated in a guided ACP conversation. An analysis of ADs, made during or following an ACP conversation, may provide insight into the various factors that are important to seriously ill patients.

The aim of this study was to get insight into the content of ADs completed by patients with advanced cancer who participated in a structured ACP conversation.

## Methods

### Research design

This study represents a sub-study of the ACTION trial, a phase III multicentre cluster randomised controlled trial that evaluates the ACTION Respecting Choices (RC) ACP intervention in patients with advanced cancer (Trial Number: ISRCTN63110516) [20]. Twenty-three hospitals in six European countries—Belgium (BE), Denmark (DK), Italy (IT), the Netherlands (NL), Slovenia (SI) and the United Kingdom (UK)—were randomised in the intervention arm (*n* = 12) (ACTION RC ACP intervention) or control arm (care as usual) *(n* = 11) [20]. The ACTION RC ACP intervention involved one or two scripted conversations between an ACTION RC ACP trained facilitator, the patient and, if the patient wishes, a person nominated as their personal representative (PR). The facilitators assisted patients during these conversations in exploring their understanding of their illness, reflecting on their goals and values and to consider their future preferences (Supplementary file 1). Additionally, facilitators encouraged patients to document their goals and preferences for future medical treatment and care in a so-called My Preferences Form (MPF) (Supplementary file 2 and 3). The MPF was developed for the ACTION trial and can be used—depending on local regulations—as an AD. This comprehensive form consisted of free text sections and tick box sections, requiring qualitative and quantitative methods of analysis, respectively. Therefore, a mixed method design was used in which qualitative and quantitative strategies were pursued in parallel [21].

### Population

Patients with advanced lung cancer (small cell—extensive disease/stage III or IV and non-small cell—stage III or IV) or colorectal cancer (stage IV or metachronous metastases) were invited by their treating healthcare professional to participate in the ACTION trial between May 2015 and December 2017. Patients were eligible to participate when they were ≥ 18 years and had a WHO performance status of ≤ 3. Patients were excluded when they had less than 3-month anticipated life expectancy or were unable to complete the questionnaire in country’s language. For this sub-study, we included all patients participating in the intervention arm of the ACTION trial who completed and returned a MPF as part of this intervention.

### Data collection

Data consisted of returned and completed MPFs. Data collection continued until 1 January 2019, 1 year after inclusion for the ACTION trial had finished.

The MPF includes information about the patient’s PR, exploratory sections regarding ‘Living well’ (section A1), ‘Worries and fears’ (section A2), ‘Beliefs’ (section A3) and ‘Hopes’ (section B), and preferences sections concerning Cardio-Pulmonary Resuscitation (CPR) (section C), goals of future care (section D), final place of care (section E) and other preferences (section F). MPFs where at least one of the six sections of the form was filled in were included for analysis.

Background data (demographic characteristics and medical conditions) were retrieved from the patients’ medical files and the facilitators’ report of the ACP conversation.

### Data analysis

The ACTION research team of each country collected and anonymised the MPFs.

The indicated preferences written in the tick boxes (sections C, D and E) were extracted and converted into an Excel document. This data was analysed by quantitative descriptive analyses using the Statistical Package for the Social Sciences (SPSS version V21.0).

To facilitate qualitative analysis, the free text sections (sections A, B and F) were translated into English by the local ACTION researchers of DK, IT and SI. Subsequently, the content of all forms was imported into an Excel document. This data was analysed using content analysis, a qualitative method to analyse text data [22]. We started with (re) reading the answers of the open sections to become familiar with the data. Subsequently, two authors both skilled in conducting qualitative research (MZ, MK) independently started with open coding of the first three MPFs of each country (15% of included MPFs). During several meetings, MZ and MK discussed the initial codes per section of the MPF, working towards intersubjective agreement. Related codes were then clustered into categories (Supplementary file 4. Code tree). MZ continued the process of coding and categorizing. MK checked coding and the interpretation of the data. Saturation was achieved, meaning that the analysis of the last included MPFs did not uncover ideas that could not be assigned to already existing categories [23]. The content analysis was supported by NVivo 11.

One researcher of each local team checked whether the reported outcomes were in line with the content of the MPFs of their country. No significant adjustments to the categories were made. Finally, relevant quotes were extracted from the MPFs to fully convey the essence of the categories.

### Ethical considerations

Ethical approval was obtained from the institutional review board (IRB) of the coordinating centre (‘Medische Ethische Toetsings Commissie (METC) Erasmus MC’), as well as IRBs in all participating countries (NL 50012.078.14). Informed consent was obtained from all individual participants in the study.

## Results

**Fig. 1:**
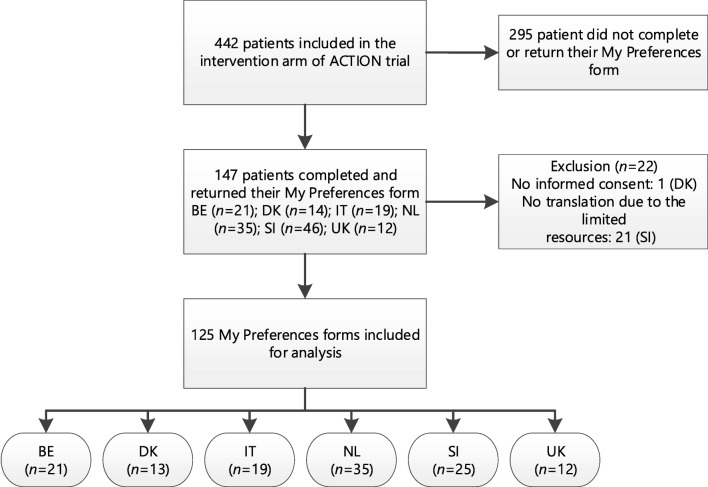
Inclusion my preferences forms for analysis

Of the 442 patients who participated in the intervention arm of the ACTION trial, 147 had returned the MPF by 1 January 2019. Document completion varied per country: 10.4% (UK), 20.6% (DK), 29.2% (BE), 41.7% (NL), 61.3% (IT) and 63.9% (SI). Of the 147 MPFs, 125 forms were included for analysis (Fig. 1). In total, 22 MPFs were excluded, mainly due to limited resources for translation (*n* = 21). One patient refused consent.

Respondents were on average 66.9 years of age, most of them were male (61.2%) and 53.6% of the patients suffered from lung cancer (Table 1). Many patients (*n* = 96) completed the MPF during the ACTION RC ACP conversation. Most patients completed at least four of the six sections (*n* = 114), including 22 patients who completed all sections.

**Table 1:** Background characteristics of patients who completed a MPF

	*N* (%)
*N* patients	125 (100%)
Male	77 (61.6%)
Age	Mean 66.9 (range 40–86)
Marital status	
Married/civil partnership	81 (64.8%)
Unmarried	11 (8.8%)
Divorced/separated	16 (12.8%)
Widowed	15 (12%)
Living with a spouse/partner	86 (68.8%)
Living in a private household	118 (94.4%)
Children, yes	114 (91.2%)
Number of children living at home	Mean 2 (range 1–3)
Total number of years of education	Mean 12.9 (Range 5–26)
Being religious	52 (41.6%)
Catholic	30 (24%)
Protestant	5 (4%)
Church of England	5 (4%)
Member of a minority ethnic group in your country	1 (0.8%)
Type of cancer	
Small cell—extensive disease lung cancer	18 (14.4%)
Non-small cell lung cancer	49 (39.2%)
Colon cancer	42 (33.6%)
Rectal cancer	14 (11.2%)
Stage of cancer	
Stage III, lung cancer	16 (12.8%)
Stage IV, lung cancer	51 (40.8%)
Colorectal cancer stage IV	44 (35.2%)
Colorectal cancer—metachronous metastases	11 (8.8%)
WHO^a^	
0	40 (32%)
1	66 (52.8%
2	14 (11.2%)
3	2 (1.6%)
Current treatment^b^	
Chemotherapy	83 (66.4%)
Radiation therapy	18 (14.4%)
Immunotherapy	4 (3.2%)
Targeted therapy	12 (9.6%)

Data are means ± range or *n* (%) of total number of patients of whom information was available, this could be different from the total *n* of 125

^a^World Health Organisation performance status scale (0—you are fully active and more or less as you were before your illness; 1—you cannot carry out heavy physical work, but can do anything else; 2—you are up and about more than half the day and can look after yourself, but are not well enough to work; 3—you are in bed or sitting in a chair for more than half the day and you need some help in looking after yourself; 4—you are in bed or a chair all the time and need a lot of looking after)

^b^Some patients received more than one treatment at the same time

Below, each section of the MPF will be presented separately in the same order these sections appear in the MPF and the percentage of patients who completed each section is provided.

### Personal representative (90%)

Of the 125 patients, 113 patients (90%) had chosen someone to make decisions on their behalf if they would become unable to make decisions themselves.

### Exploratory sections

#### Section A1 (free text): activities or experiences that are important for me to live well (94%)

‘Maintaining normal life’, ‘undertaking activities’, ‘being independent’ and ‘experiencing meaningful relationships’ were categories that appeared to be essential to live well for many patients from all participating countries.

Patients often described in their MPF ‘maintaining normal life’, for example: ‘To live a normal life, to maintain the everyday life’ (DK). It appeared that maintaining normal life enabled some patients to enjoy life.

The variety of described activities was captured in the category ‘undertaking activities’. Daily activities such as walking, gardening and engaging in hobbies were mentioned as well as special activities, such as going on holidays or activities with beloved persons. ‘A day at the sea with my loved ones’ (IT)

‘Being independent’ was described by patients in different ways. Some patients used the word ‘independent’ as such, others described for example being able to communicate, being physically independent and remaining mentally competent. ‘To be able to take care of myself’ (DK), ‘When I can do things autonomously’ (IT) and ‘Being able to think clearly’ (SI).

‘Experiencing meaningful relationships’ was by some patients described as having a ‘family life’ (DK) or ‘friendship’ (NL). Other patients elaborated on their relationships, describing visits to family and friends or engaging in activities with them, in particular with children and grandchildren. Some patients described the importance of their life having meaning, writing down for whom and how they wanted to be of meaning. For instance, by contributing to their organization as an employee or helping their children by taking care of grandchildren.

Some patients, from NL, IT and the UK specifically, described being free from pain in this section, mainly as a precondition of living well.

#### Section A2 (free text): I have the following fears or worries (74%)

Patients from all participating countries feared the consequences of disease progression. Some patients expressed this in a general way, ‘Fears and worries about the complications of the illness’ (IT), while others were more concrete in their worries and fears regarding disease progression. For example: having less energy, physical decline, hopelessness suffering and frightening experiences (e.g. ‘to be in pain’ [SI]). Several patients described their fear of becoming dependent or being in a vegetative state. As one patient expressed: ‘My greatest fear is being trapped in an unresponsive body’ (UK).

Patients also struggled with unpredictability, worrying about the outcomes of their treatment and how much time they had left. ‘Naturally, I am worried about whether the treatment will work on me’ (DK).

Becoming unable to maintain their normal life was a fear expressed by a few patients as well as the worry or fear of being taken to a final place of care they disliked.

Several patients from IT, NL and the UK worried about being or becoming a burden or causing distress to their relatives. Some patients worried about how their loved ones would recover after they had passed away. For example, one patient mentioned being married for 50 years and was worried about his spouse.

Several patients wrote that they had no worries or not yet. Others mentioned they actively avoided thinking about worries and described living 1 day at the time or trying not to think about worries and fears: ‘Of course I have fears and worries, but I will not let my life be influenced by it. ‘It goes the way it comes’ (NL).

#### Section A3 (free text): I have the following cultural, religious or spiritual beliefs (55%)

Religion was described by most patients. Fifteen patients specified their religion (e.g. Church of England, Catholic or Christian). The same number of patients elaborated on the role their religion played in their lives regarding their disease or described preferences based on their religion. For example, ‘I have no fear of dying, I know He is waiting for me’ (NL) or ‘Church of England. I would want to see the vicar if I was very poorly’ (UK).

Regarding personal beliefs, a few patients described living day-by-day, not giving up and being positive. One patient described: ‘I believe in faith, that the course of life and experiences are predestined’ (SI). Some patients addressed their belief in science and the health system. Not having any beliefs that affected their wishes was also described by a number of patients.

#### Section B (free text): my hopes for my current medical plan of care include (96%)

The majority of patients hoped for prolongation of life. Several patients expressed this as hope for a cure, remaining stable or the hope that their tumour would shrink. Other patients described prolongation of life in terms of being able to reach a special moment. ‘I hope to await my daughter’s graduation’ (BE). A few patients wanted to prolong their lives in the hope that science would make progress on treatment that improved their chance for survival.

Hope to diminish the burden of the disease was also described and included being free from suffering as well as symptom relief. Patients mentioned in particular the hope of being free from pain.

Patients from all participating countries described their hope to remain independent and expressed the hope that they would remain able to take care of themselves.

Another hope expressed by patients was being looked after by healthcare professionals. This was specified as the hope for frequent appointments and good collaboration with the healthcare professional, which included receiving clear and honest information.

Some patients shared their goals of care in the case of deterioration (NL, SI, UK). For example, ‘To a certain limit (treatment) as long as tolerant and humane to me’ (NL). Others hoped to stay at home as long as possible (BE, NL, UK) or to die with dignity (BE, IT, UK): ‘When it comes to the end, I want to go in peace and not to keep me hanging on.’ (UK).

Described hopes also included maintaining a normal life and enjoying life: ‘Hope chemo will maintain my current quality of life’ (UK). Some patients from NL, IT and SI described their state of mind in the section of hope. These patients wanted to stay positive, were willing to fight or trusted their healthcare professional. Only one patient described not having any hopes because of the advanced stage of the disease.

### Preferences sections

#### Section C (tick box): my preferences regarding resuscitation (96%)

Two thirds of the patients (*n* = 78) indicated their preference to receive CPR if their physician considered it medically appropriate in their actual situation (Table 2). This option was chosen most often in IT and DK (respectively, 89.5% and 76.9%). Eight patients explained their choice by referring to the circumstances in which they did or did not want CPR. ‘If after CPR I will return in a condition I am right now, I would choose CPR. Otherwise not’ (SI).

**Table 2:** Indicated preferences

	BE	DK	IT	NL	SI	UK	Total
	(*n* = 21)	(*n* = 13)	(*n* = 19)	(*n* = 35)	(*n* = 25)	(*n* = 12)	(*n* = 125)
Cardio pulmonary resuscitation (CPR) (section C)							
I wish to have CPR attempted if my physicianconsiders it medically appropriate in my actual situation.	11 (52.4%)	10 (76.9%)	17 (89.5%)	20 (57.1%)	13 (52%)	7 (58.3%)	78 (62.4%)
I do not wish CPR attempted if my heart or breathing stops.	9 (42.9%)	3 (23.1%)	2 (10.5%)	14 (40%)	7 (28%)	5 (41.7%)	40 (32%)
Added sentences^a^	1	4	0	1	2	0	8
Left open	1 (4.8%)	0	0	0	4 (16%)	0	5 (4%)
Goals of care (section D)							
Selective treatment plus comfort-focused care	3 (14.3%)	6 (46.2%)	17 (89.5%)	12 (36.3%)	12 (48%)	8 (66.7%)	58 (46.4%)
Comfort-focused care	13 (61.9%)	5 (38.5%)	2 (10.5%)	22 (63%)	9 (36%)	3 (25%)	54 (43.9%)
Added sentences^a^	1	3	0	8	4	2	18
Left open	5 (23.8%)	2 (15.4%)	0	0	3 (12%)	0	10 (8%)
Final place of care (section E)							
I have a preferred final place of care	16 (76.2%)	11 (84.6%)	14 (73.7%)	28 (80%)	25 (100%)	11 (91.6%)	105 (84%)
This place is^b^:							
Home	10	6	7	24	20	8	75
Hospice	3	6	3	6	0	2	20
Hospital	5	1	2	0	0	2	10
Other	0	2	3	0	5	0	9
I do not have a preferred final place of care.	4 (19.0%)	2 (15.4%	3 (15.8%)	6 (17.1%)	0	0	15 (12.2%)
Left open	1 (4.8%)	0	2 (14.3%)	1 (2.9%)	0	1 (8.3%)	5 (4%)

^a^Either added sentences to the choice made or only described information without making a choice

^**b**^Patients could write more than one preferred final place of care

#### Section D (tick box): my goals of future care (92%)

Preferences regarding goals of future care were almost equally divided between ‘Comfort-Focused Care’ and ‘Selective Treatment plus Comfort-Focused Care’ (Table 2). In NL and BE, the majority of the patients preferred ‘Comfort-Focused Care’. In other countries, the majority of the patients chose ‘Selective Treatment plus Comfort-Focused Care’, where the primary goal is treating a complication. All Italian respondents, except for two, chose the latter option.

A few patients precisely articulated what they meant by their preferences. For example: ‘Would like to have for example IV antibiotics, if it seems to have an effect and it is only for a short period of time. Do not wish to be treated for infections if the illness is much progressed and it is futile’ (DK).

#### Section E (tick box): my preferences regarding final place of care (96%)

In all six countries, the vast majority of patients reported a preferred final place of care (84%), most often ‘home’ (*n* = 75) (Table 2). Others preferred a hospice (*n* = 20) or hospital (*n* = 10). Patients who added specific information (*n* = 24) mainly specified personal aspects of quality ‘[living] at home with family’ (IT), ‘[living] as long as possible and in a good condition’ (BE) or ‘with a view to my garden’ (NL). A few patients added what they did not want. ‘Hospice/hospital. Not home’ (UK).

#### Section F (free text): my other preferences that I consider important to be known by those who care for me (41%)

Most patients used this section to add explanations following the information provided in one of the previous sections of the MPF. To illustrate: ‘If causing distress to family or if unable to be treated at home, I would like my personal representative to decide if a nursing home, hospital or hospice is the best alternative’ (UK).

A few patients wrote down preferences regarding their wish for alternative treatment, or to prevent futile treatment. A wish for euthanasia in the case of unbearable suffering was reported by a few patients from NL and BE.

Preferences regarding the stage of deterioration and dying were also mentioned, including wishes about visitors and family (‘I wish that not too many people will visit at one time’ [DK]) and being free from pain.

After-death arrangements were also described by several patients. Some patients shared their preferences regarding their funeral (e.g. cremation and pictures on the coffin) or organ donation.

## Discussion

We found that one third of patients participating in an ACP intervention completed an AD. The degree of completion varied substantially between countries. Analysis of ADs showed that the topics described by patients in the exploratory sections mainly concerned maintaining a normal life, hope for prolonging life and experiencing meaningful relationships. Also, the fear of suffering from disease progression and becoming dependent was often described. Most patients chose a PR and preferred ‘home’ as their final place of care. Preferences regarding CPR and goals of future care varied between patients and countries.

In the exploratory sections, many patients described their values, wishes and hopes, as well as their fears or worries in a rather concrete way. Similar to a study by Trarieux-Signol et al. (2018) [19], who predominantly analysed blank sheet ADs, we found that preventing functional and mental dependency, effective symptom treatment and after-death arrangements were considered important [19]. However, it seemed that patients in our study provided more information regarding worries, fears and hopes. To illustrate, patients not only formulated their hope to prolong life, but also their hope that science would make progress to improve their chances for prolonging their lives. It is likely that patients provided more information because they were asked specifically about this during the ACP conversation. Studies investigating hope in palliative care confirm this variety in objectives, meanings and functions of hope [24–26].

A completed AD with such broad information might provide healthcare professionals and relatives with a better insight into the patients’ perspectives and might improve the guidance of the professionals throughout the end of life process when applying the AD. However, previous studies described the importance of ADs being as precise as possible and that ADs should include relevant information for healthcare professionals to make decisions [19, 27, 28]. Other ADs often prompt patients to indicate preferences concerning specific life-prolonging treatments [4, 17, 18]. It is known that patients may find it difficult to complete such ADs [16, 19, 29]. In contrast, the preferences sections of the MPF in our study contained two sections that formulated preferences in a broader way, e.g. ‘goals of future care’ and ‘other preferences’. These sections shed light on the patients’ goals and intentions with respect to medical treatment and care. Although less specific, it might be easier for patients to indicate their perspectives and preferences this way, which could result in an increased completion of ADs. This highlights the need for a conversation between the patient and their healthcare professional in order to gain a better understanding of the patient’s expressed preferences in an AD, and to suggest and share thoughts on medical treatments that align to the patient’s values.

It is important to be aware of some limitations of this study. We included forms of patients who might be more open to completing a form or who completed the form during the conversation. This might have influenced the results of this study. In addition, only one patient was a member of a minority ethnic group, this might limit the extent to which these results can be applied to other populations. Additionally, although translated carefully, some information or nuances may have been lost in translation. However, by validating the results with native speaking researchers of each participating country, we believe that we took sufficient measure to mitigate this limitation.

In conclusion, this study provides the insight that being independent, maintaining a normal life, having meaningful relations and being free from pain are important topics in ADs for patients with advanced cancer in Europe. A more comprehensive AD, meaning an AD that includes exploratory sections and preferences, provides healthcare professional and relatives a better perspective of the most important values of patients at the end of their life, and, therefore, offers an opportunity to improve the guidance of the healthcare professional. Having a conversation to understand the reasoning behind indicated preferences remains essential for relatives and healthcare professionals to make decisions that are in line with the preferences of the patient.

## Electronic supplementary material

ESM 1 (PDF 469 kb)

ESM 2 (PDF 670 kb)

ESM 3 (PDF 320 kb)

ESM 4 (PDF 223 kb)
