# Supplementary material for: Content analysis of Advance Directives completed by patients with advanced cancer as part of an Advance Care Planning intervention: insights gained from the ACTION trial
Source: Support Care Cancer. 2019 Jul 5;28(3):1513–22. doi: 10.1007/s00520-019-04956-1 (PMC6989617; doi:10.1007/s00520-019-04956-1)
Supplement: Supplementary file 2 — (PDF 670 kb) [file 520_2019_4956_MOESM2_ESM.pdf]

Article title: Content analysis of Advance Directives completed by patients with advanced cancer as part of an Advance Care Planning intervention: insights gained from the ACTION trial

Journal name: Journal of supportive care in cancer

Author names; M. Zwakman, J.J.M. van Delden, G. Caswell, C.A. Christensen, L. Deliens, F. Ingravallo, L.J. Jabbarian, A.T. Johnsen, I. J. Korfage, A. Mimić, N.J. Preston, M.C. Kars. On behalf of the ACTION consortium.

Affiliation of the corresponding author:
Julius Center for Health Sciences and Primary Care, University Medical Center Utrecht, the Netherlands

E-mail address of the corresponding author:
m.zwakman@umcutrecht.nl

ACTION

My Preferences

ACTION is an international study about advance care planning in which patients are supported by a trained facilitator to express their preferences for care and treatment.

This My Preferences form has been constructed in the context of this ACTION study. The form is a communication tool. It describes goals and preferences concerning medical treatment and care of

……………………………………………………………………………………………. *(insert name).*

Please note that this form is not legally binding but contains useful information for all involved in the patient's care. *[this sentence needs to be adapted to local circumstances]*

More information about the ACTION study can be found at www.action-acp.eu.

Contact information for the ACTION project
Name
Email address
Tel.

This My Preferences form is a **communication tool** which describes goals and preferences in relation to future treatment and care.

[appropriate logo's]

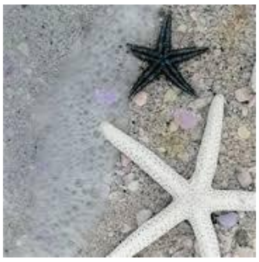

My Preferences

THIS IS THE MY PREFERENCES FORM OF:

First name and last name:

Date of birth: __ (day) - __ (month) – ____ (year) Gender: Male / Female

Place of birth:

Address:

Tel. number:

INFORMATION ABOUT MY PERSONAL REPRESENTATIVE:

My Personal Representative is the person I have asked to express my preferences for me so that they can be taken into account should I at some point be unable to make my own decisions.

First name and last name:

Date of birth: __ (day) - __ (month) – ____ (year) Gender: Male / Female

Address:

Tel. number:

If applicable:

This My Preferences form is a **communication tool** which describes goals and preferences in relation to future treatment and care.

☐I do not have a Personal Representative

Section **A**<br><br>My thoughts about 'living well'	❖ Activities or experiences that are important for me to live well:<br><br>❖ I have the following fears or worries:<br><br>❖ I have the following cultural, religious or spiritual beliefs:
Section **B**<br><br>My hopes for my current medical plan of care	❖ My hopes for my current medical plan of care include:

This My Preferences form is a **communication tool** which describes goals and preferences in relation to future treatment and care.

If I were to become unable to communicate and express my preferences I would like the following issues to be taken into account (sections C-F):

Section **C**<br><br>My preferences regarding resuscitation	*(Initial one choice below)*<br><br>_____ I wish to have CPR attempted if my physician considers it medically appropriate in my actual situation.<br><br>_____ I do not wish CPR attempted if my heart or breathing stops.

This My Preferences form is a **communication tool** which describes goals and preferences in relation to future treatment and care.

<table>
<tr>
<td>Section
D

My goals of future care</td>
<td>I know that it happens to some patients that they experience a potentially life-threatening complication. Should I have such a complication while I am unable to communicate and express my preferences, the following option best fits my goals and values:
(Initial one choice below)

______ **Selective Treatment plus Comfort-Focused Care**
Primary goal of attempting to treat the complication
I would like my physician to provide me with interventions he thinks might help me recover from the complication and extend my life. In addition to that, pain and suffering will be relieved. This may include medications and other simple interventions to treat any symptoms I may have. I understand that any of these treatments may involve transfer to hospital.

______ **Comfort-Focused Care**
Primary goal of maximizing comfort.
The focus of this option is to relieve pain and suffering. This may include medications and other simple interventions to treat any symptoms I may have. I understand that this may only involve transfer to hospital if my comfort needs cannot be met in my current location.

(Clarify or elaborate below, if appropriate)</td>
</tr>
<tr>
<td>Section
E

My preferences regarding final place of care</td>
<td>*(Initial one choice below)*

______ I have a preferred final place of care.
This place is: *(please insert)*

______ I do not have a preferred final place of care.</td>
</tr>
</table>

This My Preferences form is a **communication tool** which describes goals and preferences in relation to future treatment and care.

Section **F** My other preferences that I consider important to be known by those who care for me	
My signature (required)	Place and date signed
Signature of my Personal representative (preferably)	Place and date signed

(NO signature of medical doctor is needed as this form is a communication tool)

☐ I have also completed a legally valid document about my health care preferences.*

☐ I plan to complete a legally valid document about my health care preferences.*

☐ I do NOT plan to complete a legally valid document about my health care preferences.*

** Tick one box*

This My Preferences form is a **communication tool** which describes goals and preferences in relation to future treatment and care.

Information for those who completed this My Preferences form and their carers

- We encourage you to discuss this form with your doctor(s) and caregivers.
- If you wish, you can hand (a copy of) this form over to your doctor(s) and caregivers.
- We recommend you to review this form periodically. We also recommend you to review it if your preferences change or when there is a substantial change in your health status.
- If you have reviewed your form and do NOT want to make changes, please indicate so below, add the date and add your signature.
- If you have reviewed your form and want to complete a new form, you can destroy your earlier form. You have the following options to get a new form:
    - Form can be downloaded from www.action-acp.eu
    - You can phone or email the contact person from the research team, details are listed at the first page of this document.
    - You can use the extra copy that was provided by the facilitator.
- If you have updated this form we encourage you to discuss this with your doctor(s) and caregivers, and anyone else you might have discussed your preferences with.

Refer to legal documents if applicable [please provide country-specific details].

This My Preferences form is a **communication tool** which describes goals and preferences in relation to future treatment and care.

This form will be stored in an electronic database for research purposes. We will add a study number to this form. We will remove all personal details of you and of your Personal Representative.

If you have reviewed your form and do NOT want to make changes, please indicate this below.

☐ I have reviewed this form on ……………………… *(date)*

I confirm my indicated preferences.

My signature (required)

☐ I have reviewed this form on ……………………… *(date)*

I confirm my indicated preferences.

My signature (required)

☐ I have reviewed this form on ……………………… *(date)*

I confirm my indicated preferences.

My signature (required)
