# Supplementary material for: Content analysis of Advance Directives completed by patients with advanced cancer as part of an Advance Care Planning intervention: insights gained from the ACTION trial
Source: Support Care Cancer. 2019 Jul 5;28(3):1513–22. doi: 10.1007/s00520-019-04956-1 (PMC6989617; doi:10.1007/s00520-019-04956-1)
Supplement: Supplementary file 3 — (PDF 320 kb) [file 520_2019_4956_MOESM3_ESM.pdf]

Article title: Content analysis of Advance Directives completed by patients with advanced cancer as part of an Advance Care Planning intervention: insights gained from the ACTION trial

Journal name: Journal of supportive care in cancer

Author names; M. Zwakman, J.J.M. van Delden, G. Caswell, C.A. Christensen, L. Deliens, F. Ingravallo, L.J. Jabbarian, A.T. Johnsen, I. J. Korfage, A. Mimić, N.J. Preston, M.C. Kars. On behalf of the ACTION consortium.

Affiliation of the corresponding author:

Julius Center for Health Sciences and Primary Care, University Medical Center Utrecht, the Netherlands

E-mail address of the corresponding author:

m.zwakman@umcutrecht.nl

Supplementary file 3. Information of the My Preferences Form

The My Preferences form was developed by the ACTION research team for the purposes of the ACTION trial. The form can be seen and used –depending on local regulations– as an Advance Directive. Aside from the legal status of the My Preferences form, it can provide useful information for both healthcare professionals and family members when they have to make a decision on behalf of the patient.

The My Preferences form consists of six sections with open and closed questions concerning patients' wishes and preferences in relation to their future medical treatment and care. The first two sections (section A and B) are open sections and explorative. In these two sections, the patients can describe their thoughts regarding 'Living well' (section A), 'Worries and fears' (section A), 'Beliefs' (section A), and 'Hopes' (section B).

The next three sections (section C, D, and E) are multiple choice questions and consist of decisions regarding the (non-)use of potentially burdensome life-prolonging interventions. In section C, Cardio-Pulmonary Resuscitation (CPR), the patient can choose between (1) 'I wish to have CPR attempted if my physician considers it medically appropriate in my actual situation' or (2) 'I do not wish CPR attempted if my heart or breathing stops'. In section D, goals of future care, the patient can choose between 'Selective Treatment plus Comfort-Focused Care' (Primary goal of attempting to treat the complication) and 'Comfort-Focused Care' (Primary goal of maximizing comfort). Both sections are closed, but patients are able to included additional information regarding their preference. In section E, the patients can write whether they have a preferred final place of care. If yes, they are able to describe which place (more answers are possible).

In the last section (section F) there is space for patients to include other information which the patients consider as important to share with relevant others.

Patients have the opportunity to complete this form during the ACTION RC ACP conversation with the facilitator, but may also complete the form at home, at their own convenience.
