# Supplementary material for: Content analysis of Advance Directives completed by patients with advanced cancer as part of an Advance Care Planning intervention: insights gained from the ACTION trial
Source: Support Care Cancer. 2019 Jul 5;28(3):1513–22. doi: 10.1007/s00520-019-04956-1 (PMC6989617; doi:10.1007/s00520-019-04956-1)
Supplement: Supplementary file 4 — (PDF 223 kb) [file 520_2019_4956_MOESM4_ESM.pdf]

Article title: Content analysis of Advance Directives completed by patients with advanced cancer as part of an Advance Care Planning intervention: insights gained from the ACTION trial

Journal name: Journal of supportive care in cancer

Author names; M. Zwakman, J.J.M. van Delden, G. Caswell, C.A. Christensen, L. Deliens, F. Ingravallo, L.J. Jabbarian, A.T. Johnsen, I. J. Korfage, A. Mimić, N.J. Preston, M.C. Kars. On behalf of the ACTION consortium.

Affiliation of the corresponding author:

Julius Center for Health Sciences and Primary Care, University Medical Center Utrecht, the Netherlands

E-mail address of the corresponding author:

m.zwakman@umcutrecht.nl

Supplementary 2. Code tree

	Category	Code	Subcode
**Section A: living well**			
	Maintaining normal life		
		Keeping the daily routine	
		Feeling healthy	
		Enjoying life	
	Undertaking activities		
		Daily activities	
			Gardening
			Walking
			Shopping
			Hobby
			Eating & drinking
		Special activities	
	Being independent		Holiday
		Being able to communicate	
		Remain mentally competent	
		Remain physically independent	
	Experiencing meaningful relationships		
		Family	
		Friends	
		Being of meaning	
			To other people
			Work
	Being free from pain		
	Additional aspects of living well		
		Nature	
		No worries, peace, no stress	
**Section A: Worries and fears**			
	Patient Worries		
		Disease progression	
			Physical decline
			A fear of frightening moments
			Hopeless suffering
			Being dependent
			Being in a vegetative state
		Unpredictability	
			Effect treatment
			Time left

		Final place of care	
		Unable to maintain normal life	
	Worries about loved ones		
		Worries about relatives	
		Being a burden	
	No worries		
	Additional worries/fears		
**Section A: Beliefs**			
	Religious beliefs		
		Value	
		Take into account	
		Atheist	
	Personal beliefs		
	Spiritual beliefs		
	No beliefs		
	Additional information		
**Section B: Hopes**			
	Prolonging life		
		Cure	
		Miracle	
		Shrink of the tumor	
		Being stable	
		Maintain/improve physical condition	
		Being able to be present at a special moment	
		Benefit from new treatment	
	Burden of disease		
		Relieve symptoms	
		No suffering	
	Preserving independency		
		Staying mentally competent	
		Being able to communicate	
	Being looked after		
		Communication with health care professionals	
		Appointments with health care professionals	
	In case of deterioration		
		Goals of care	
		Place of care	
		Dying with dignity	
	Quality of life		
		Maintaining normal life	
		Enjoying life	
		Family	
	State of mind		

		Trust in doctors	
		Staying positive	
		Fighting	
	No hope		
	Additional hopes		
**Section F: My other preferences that I consider important to be known by those who care for me**			
	Additional information to section A, B, C, D or E		
	Treatment		
		No endless treatment	
		Alternative medicine	
		Euthanasia	
	Stage of deterioration and dying		
		Visits	
		Family	
		Dignity	
	After-death arrangements		
		Funeral	
			Giving substance to the funeral
			Decision about the location of the funeral
		Organ donation	
	No other preferences		
	Additional preferences		
